# Comparative Molecular Field Analysis (CoMFA) and Comparative Molecular Similarity Indices Analysis (CoMSIA) Studies on α_1A_-Adrenergic Receptor Antagonists Based on Pharmacophore Molecular Alignment

**DOI:** 10.3390/ijms12107022

**Published:** 2011-10-20

**Authors:** Xin Zhao, Minsheng Chen, Biyun Huang, Hong Ji, Mu Yuan

**Affiliations:** 1 Pharmaceutical Research Center, Guangzhou Medical College, Guangzhou, Guangdong 510182, China; E-Mails: zhaoxin.gy@gmail.com (X.Z.); gzminsheng@vip.163.com (M.C.); guangyihby@126.com (B.H.); dljih@126.com (H.J.); 2 Department of Food and Bioengineering, Guangdong Industry Technical College, Guangzhou, Guangdong 510300, China

**Keywords:** CoMFA, CoMSIA, pharmacophore-based molecular alignment, α_1A_-adrenoceptor antagonists, GALAHAD, activity prediction

## Abstract

The α_1A_-adrenergic receptor (α_1A_-AR) antagonist is useful in treating benign prostatic hyperplasia, lower urinary tract symptoms, and cardiac arrhythmia. Three-dimensional quantitative structure-activity relationship (3D-QSAR) studies were performed on a set of α_1A_-AR antagonists of N-aryl and N-nitrogen class. Statistically significant models constructed from comparative molecular field analysis (CoMFA) and comparative molecular similarity indices analysis (CoMSIA) were established based on a training set of 32 ligands using pharmacophore-based molecular alignment. The leave-oneout cross-validation correlation coefficients were *q*^2^ _CoMFA_ = 0.840 and *q*^2^ _CoMSIA_ = 0.840. The high correlation between the cross-validated/predicted and experimental activities of a test set of 12 ligands revealed that the CoMFA and CoMSIA models were robust (*r^2^* *_pred_*_/CoMFA_ = 0.694; *r^2^* *_pred_*_/CoMSIA_ = 0.671). The generated models suggested that electrostatic, hydrophobic, and hydrogen bonding interactions play important roles between ligands and receptors in the active site. Our study serves as a guide for further experimental investigations on the synthesis of new compounds. Structural modifications based on the present 3D-QSAR results may lead to the discovery of other α_1A_-AR antagonists.

## 1. Introduction

Adrenergic receptors are members of the G-protein coupled receptor superfamily of membrane proteins that mediate effects of the sympathetic nervous system through the actions of epinephrine and norepinephrine and control the equilibrium of the cardiovascular system. The receptors have been classified into three classes (α_1_, α_2_, and β) with three members in the α_1_ subfamily, namely, α_1A_, α_1B_, and α_1D_ [1]. Many physiological processes, including smooth muscle contraction, myocardial inotropy and chronotropy, and hepatic glucose metabolism are related to the α_1A_-adrenergic receptors (α_1A_-ARs); thus, clinical applications of these receptors are of interest to many scientists. The α_1A_-AR antagonists are considered as the first-line of therapy for lower urinary tract symptoms associated with clinical benign prostatic hyperplasia (BPH), because they have proven efficacy in mediating smooth muscle contraction in the human prostate [2].

Reports on the application of QSAR analysis to α_1_-AR species are relatively limited [3–6]. The SAR of 39 α_1_ adrenoceptor antagonists derived from the antipsychotic sertindole with respect to affinity was investigated using a 3D-QSAR approach based on the GRID/GOLPE methodology by Balle’s group [7]. Montorsi reported quantitative size and shape affinity/subtype selectivity relationships derived from a large set of very heterogeneous α_1a_-, α_1b_-, and α_1d_-adrenergic receptor antagonists [8]. Maciejewska *et al*. reported a structure-activity analysis of hexahydro and octahydropyrido-(1,2-c)-pyrimidine derivatives as α_1A_-AR antagonists, using comparative molecular field analysis (CoMFA) [9]. Li’s and Xia’s groups provided insights into α_1A_-adrenoceptor antagonists through self-organizing molecular field analysis in the same year [10]. Shakya *et al*. reported 3D-QSAR models based on a series of 25 dihydropyridine class compounds using the APEX-3D program, which can automatically identify biphore (pharmacophore) sites, and 3D-QSAR modeling [11]. Compared with SOMFA studies, the model has strong predictability, particularly for compounds with enantio-selectivity. Last year, Gupta reported on studies based on CoMFA and comparative molecular similarity indices analysis (CoMSIA) performed on a set of diverse α_1A_-AR antagonists to understand the relationship between structure and antagonistic activity [12]. This study was based on common structural alignments. The generated models suggest that steric, electrostatic, and hydrophobic interactions play important roles in structure-activity analysis.

The superimposition of compounds in an alignment is crucial in 3D-QSAR studies. Many studies have reported that pharmacophore alignment is a useful tool that is superior to others [13,14]. Genetic algorithm with linear assignment of hypermolecular alignment of datasets (GALAHAD^®^) is regarded as a superior tool for molecular alignment compared with classical common structural alignment [15], especially for compounds that share few structural commonalities. GALAHAD uses the proprietary technology of Tripos^®^ and generates pharmacophore alignments and hypotheses from sets of ligand molecules using a genetic algorithm [16]. The relationship between the chemical structures and the biological function studies based on the pharmacophore alignments of α_1A_-adrenergic receptor using GALAHAD has yet to be reported. Based on the alignment obtained, CoMFA and CoMSIA models were developed. The information derived from these models will be helpful in predicting the activity and guiding the design of other α_1A_-AR antagonists.

## 2. Materials and Method

### 2.1. Data Collection

**Figure 1:**
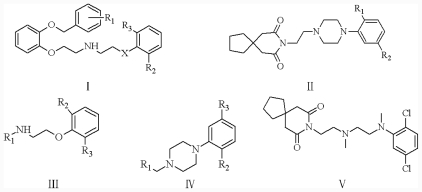
Structures of the α_1A_-AR antagonists used in the 3D-QSAR study.

All α_1A_-AR antagonist structures were collected from recent reports (Figure 1 and Table 1) [17–25]. The selected compounds have diverse structural features and cover a wide range of biological activity, spanning over four orders of magnitude (0.1–630 nM). The training and test sets were classified to ensure that both sets could completely cover the whole range of biological activity and physicochemical and structural diversity studied. The need for models to be tested on a sufficiently large test set (25%–33% of the total samples) to establish a reliable QSAR model had been previously suggested [26]. Therefore, the dataset, which was composed of 44 compounds, was divided into training (32 compounds) and test (12 compounds) sets. All data from various references were from the same laboratory. The affinity constants of compounds were evaluated by radio-receptor binding assays. [*^3^H*] Prazosin was used to label cloned human α_1_-ARs expressed in CHO cells and WB4101 was used as a reference compound. *K_i_* values were derived from IC50 using the Cheng-Prusoff equation [27]. All *K_i_* values were expressed as p*K_i_* values (−log *K_i_*).

### 2.2. Structural Sketch and Alignment

Structural sketches and refinement of the entire set of α_1A_-AR antagonists were accomplished using SYBYL^®^ 8.1 molecular modeling software (Tripos Associates Inc.) and their 3D structures were generated using CONCORD^®^. All compounds were minimized under the Tripos standard (TS) force field [28] with Gasteiger-Hückel atomic partial charges [29]. Minimizations were done using the Powell method, in which calculations were set to terminate at an energy gradient value of 0.01 kcal/mol.

Pharmacophore results for N-Aryl and N-heteroaryl piperazine α_1A_-AR antagonists using GALAHAD have been reported previously [30], and the optimized model from the previous report was used as a template in the present study. All compounds in the training and test sets were aligned with this template using the “Align Molecules to Template Individually” option in GALAHAD. The other parameters for calculation were set to default values.

### 2.3. 3D-QSAR Studies

CoMFA, a method that reflects the non-bonding interaction between the receptor and the ligand, is widely used in drug design. The steric (Lennard-Jones) and electrostatic (Coulombic) potential energies of the TS force fields implemented in SYBYL were evaluated by CoMFA. For each pharmacophore alignment ligand, a 3D cubic lattice with a grid spacing of 1.0 Å in the x, y, and z directions was generated to enclose the molecule aggregate. A sp*^3^* carbon atom with a charge of +1.0 and a Van der Waals radius of 1.52 Å was used as a probe; this atom was placed at every lattice point to calculate various steric and electrostatic fields. An energy cut off value of 30 kcal/mol was imposed on all CoMFA calculations to avoid excessively high and unrealistic energy values within the molecule. Then, partial least-squares (PLS) analysis was applied to obtain the final model [31].

During calculation of the steric and electrostatic fields in CoMFA, many grid points on the molecular surface were ignored due to the rapid increase in Van der Waals repulsion. To avoid a drastic change in the potential energy of the grid points near the molecular surface, CoMSIA employed a Gaussian-type function based on distance. Thus, CoMSIA may be capable of obtaining more stable models than CoMFA in 3D-QSAR studies [31–33]. The constructed CoMSIA model provided information on steric, electrostatic, hydrophobic, hydrogen bond donor, and hydrogen bond acceptor fields.

The grid constructed for the CoMFA field calculation was also used for the CoMSIA field calculation [32]. Five physico-chemical properties (electrostatic, steric, hydrophobic, and hydrogen bond donor and acceptor) were evaluated using a common probe atom placed within a 3D grid. A probe atom sp^3^ carbon with a charge, hydrophobic interaction, and hydrogen-bond donor and acceptor properties of +1.0 was placed at every grid point to measure the electrostatic, steric, hydrophobic, and hydrogen bond donor or acceptor field. Similar to CoMFA, the grid was extended beyond the molecular dimensions by 1.0 Å in three dimensions and the spacing between probe points within the grid was set to 1.0 Å. Different from the CoMFA, a Gaussian-type distance dependence of physicochemical properties (attenuation factor of 0.3) was assumed in the CoMSIA calculation.

The partial least squares (PLS) method was used to explore a linear correlation between the CoMFA and CoMSIA fields and the biological activity values [34]. It was performed in two stages. First, cross-validation analysis was done to determine the number of components to be used. This was performed using the leave-one-out (LOO) method to obtain the optimum number of components and the corresponding cross-validation coefficient, *q^2^* [35]. The value of *q^2^* that resulted in a minimal number of components and the lowest cross-validated standard error of estimate (*S_cv_*) was accepted. The column filtering values (σ_min_) was set to 2.0 kcal/mol in order to speed up the analytical process and reduce noise. Second, the optimum number of components were used to derive the final PLS model, with no validation method [36,37]. The CoMFA and CoMSIA results were then graphically interpreted by field contribution maps.

### 2.4. Predictive Power of the Models

The predictive power of the 3D-QSAR model was determined from a set of 12 compounds in the test set (Table 2). These molecules were superimposed using the same pharmacophore molecular alignment method described above, and their activities were predicted using the CoMFA and CoMSIA models generated by the training set. The predictive correlation coefficient, *r^2^* *_pred_*, of the CoMFA and CoMSIA models were calculated using the test set, according to the formula,

(1) $$r_{pred}^{2}=\frac{\text{SD-Press}}{\text{SD}}$$

where SD is the sum of the squared deviations of each experimental value from the mean, and PRESS is the sum of the squared differences between the predicted and actual affinity values for every molecule [31].

## 3. Results and Discussion

### 3.1. Alignment

**Figure 2:**
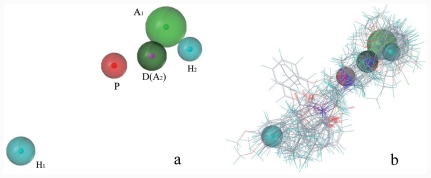
The alignment of the molecules in the present study (**b**) based on the pharmacophore hypothesis (**a**) using GALAHAD; magenta, hydrogen bond donor atom (D); green, acceptor atom (A); cyan, hydrophobic center (H); red, positive nitrogen (P).

The pharmacophore model generated by GALAHAD contained one donor center, one positive charged nitrogen atom, two acceptor centers, and two hydrophobic centers, more than other reported models, which is shown in [Figure 2(a)]. The internal distance of the pharmacophore feature is consistent with previously reported models. The pharmacophore model has reasonable assessment parameters, such as a high specificity value (4.212), high steric score (4903.5), and low energy value (92.9 Kcal/mol) [30,38]. All of the compounds were superimposed based on the template [Figure 2(b)]. Ligands have the same binding site and share common interactions. However, they sometimes do not share common structures. GALAHAD, as an alignment tool, considers the flexibility of each compound and performs a compact alignment based on essential structural elements related to activity by variation of the torsional degrees of freedom. Compared to the QSAR model previously reported, the classical approach based on a rigid alignment of minimized structures, does not show any uniformity with the active conformation. Pharmacophore-based molecular alignment using GALAHAD in the current manuscript will generate a rational alignment for 3D-QSAR studies.

### 3.2. CoMFA and CoMSIA Models

A statistically significant 3D-QSAR model was obtained using the properly selected training set of 32 ligands. Results of the statistical analysis are presented in Table 3.

In the CoMFA model, initial PLS analysis of the aligned training set was done using a default σ_min_ data filter of 2.0 kcal/mol and the Tripos standard field. This yielded a highly significant *q^2^* value of 0.840 (with *S_cv_* = 0.476, using four components), which indicates that it is a model with high statistical significance; a *q*^2^ value of 0.6 is considered statistically significant in CoMFA and CoMSIA studies [39]. The conventional *r*^2^ value of 0.988 and low standard error of estimate (SEE) value of 0.128 indicate the accuracy of the predictions of the model. High values of *q*^2^ from the LOO analysis can be regarded as a necessary, but not a sufficient, condition for a model to possess significant predictive power [40]. In addition to LOO, the internal predictive ability of the model was further assessed by a group cross-validation performed with 30 groups for 30 times. The mean of 30 readings was given as *r*^2^ _cv_; the mean *r*^2^ _cv_ of 0.837 also suggests that the model has good internal predictability.

Similar to CoMFA, the CoMSIA model with the steric, electrostatic, hydrophobic, donor and acceptor fields result in satisfactory statistical data (Table 3, model A). However, the steric and donor fields (10% and 18% contribution, respectively) did not significantly contribute to affinity in the all-fields model. Moreover, presumably correlated fields increase the complexity of contour maps and complicate their interpretation. Therefore, CoMSIA approaches with smaller subsets of fields were calculated (Table 3, columns B-E). The hydrophobic field was always considered in accordance with the hydrophobic groups in the pharmacophore model. The internal predictivities of the field combinations B-E were only slightly reduced compared to the all-fields model A. Among the five models, the combination of the electrostatic, hydrophobic, and acceptor field (model E) has the highest external predictivity (*r*^2^ _pred_ = 0.671). The conventional non-cross validated *r*^2^ of 0.975 and the SEE value of 0.180 indicate that this model is statistically highly significant. Analogous to the CoMFA, a group cross-validation was done to assess further the internal predictive ability of the model. The cross-validation was performed 30 times with 30 groups. The mean *r*^2^ _cv_ obtained was 0.864, which also indicated the robustness of this model.

### 3.3. Predictive Power of the Models

The cross-validated p*K_i_* values calculated by CoMFA and CoMSIA, and the residuals between the experimental and cross-validated p*K_i_* values of the compounds in the training set are listed in Table 4. The predictive powers of the CoMFA and CoMSIA models were further examined using a test set of 12 compounds not included in the training set. The predicted p*K_i_* values calculated by CoMFA and CoMSIA are also shown in Table 4.

**Figure 3:**
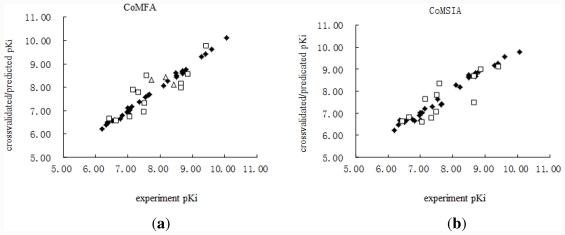
Correlation between cross-validated/predicted p*K_i_* *versus* experimental p*K_i_* for the training set (shaded diamonds) and the test set (open squares); CoMFA graph (**a**) and CoMSIA graph (**b**).

The results show that the CoMFA model (*r^2^* *_pred_* = 0.694) gives a better prediction than the CoMSIA model does (*r^2^* *_pred_* = 0.671). Plots of the cross-validated/predicted p*K_i_* *versus* the experimental values are shown in Figure 3. The shaded diamonds and open squares represent the training set and the test set, respectively.

### 3.4. Graphical Interpretation of the Fields

The CoMFA and CoMSIA contour maps of the PLS regression coefficients at each region grid point provide a graphical visualization of the various field contributions, which can explain the differences in the biological activities of each compound. These contour maps were generated using various field types of StDev*coefficients to show the favorable and unfavorable interactions between ligands and receptors in the active site.

**Figure 4:**
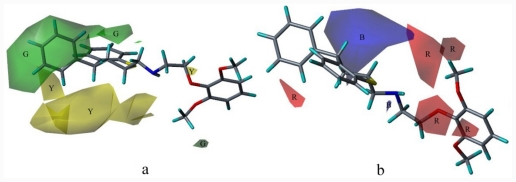
Steric (**a**) and electrostatic (**b**) contours with high-affinity compound **20** in the final CoMFA model; B, blue; G, green; R, red; Y, yellow.

In the CoMFA model, the fractions of steric and electrostatic fields are 46.0% and 54.0%, respectively. Favorable and unfavorable cutoff energies were set at the 80th and 20th percentiles for the steric contributions. The contour maps of the fields are shown in [Figure 4(a)], with the higher affinity compound **20** as the reference structure. The surfaces indicate the regions where the increase (green region) or decrease (yellow region) in steric effect would be important for the improvement of binding affinity. The large green isopleths upon the thiochromene part reflect a sharp increase in affinity for all the anchor moieties transferred into this area. Compound **20**, with its large bulky phenyl group, coincide with the green isopleths. When the thiochromene group in compound **20** was replaced by 8-methyl-8-azaspiro decane-7, 9-dione (such as compounds **24** and **32**), and the yellow region was occupied by the large bulky groups, and the antagonistic activity of these compounds evidently decreased. This was probably due to the insufficient space to accommodate these bulky groups in the anchor moieties ofthe receptor-binding site, which caused collision among groups and the reduced affinity of the compounds. [Figure 4(b)] shows the electrostatic contributions with compound **20** as a template ligand. The electrostatic contour map shows regions of red polyhedra (contribution level: 15%), where electron-rich substituents are beneficial for the binding affinity, whereas the blue colored regions (contribution level: 85%) show the areas where positively charged groups enhance the antagonistic activity. The large blue area near the thiochromane moiety indicates a region where negatively charged groups decrease antagonistic activity. Compound **32** has a carbonyl in the blue area, which may be not conducive to the improvement of activity. The electrostatic contour map shows a region of red contours neighbor to the oxygens connects with benzene, indicating that electron-rich substituents (such as bromine, cyano group) are beneficial for the binding affinity.

**Figure 5:**
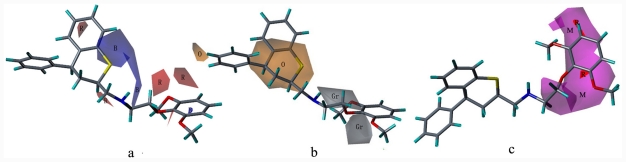
Contour plots illustrating, electrostatic (**a**), hydrophobic (**b**) and hydrogen bond acceptor (**c**) properties revealed by the CoMSIA model; high affinity compounds **20** shown as templates; B, blue; C, cyan; Gr, gray; M, magenta; O, orange; R, red.

In the CoMSIA model, the fractions of the electrostatic, hydrophobic, and hydrogen-bond donor and acceptor fields were 34.7%, 39.9% and 25.4%, respectively. The CoMSIA contour plots are also exemplified by some ligands of high affinity. Similarly, red and blue isopleths (contribution levels: 15% and 85%, respectively) of the electrostatic fields [Figure 5(a)] enclose regions, where negative and positive charges have favorable effects on *pK_i_*, respectively. The contour maps of electrostatic fields are similar to that of the CoMFA. Compared with the CoMFA model, its electronic field areas are smaller. The blue contours rather indicate where negatively charged substructures are unfavorable, such as methyl substitution and amino substitution should be placed to enhanced binding affinity, while the red contours neighboring to oxygens indicate electronegative groups will do a favorable effect of antagonistic potency.

For hydrophobic field contributions, orange and gray isopleths are drawn at contribution levels of 85% and 15%, respectively. These enclose regions favorable for hydrophobic and hydrophilic groups, respectively [Figure 5(b)]. Orange areas are mainly distributed in the thiochromene side, which is consistent with the hydrophobic center of the pharmacophore hypotheses, as well as with the contour maps of the steric fields generated by the CoMFA and CoMSIA models. Hydrophobic interactions may be dominant for ligand binding. These areas suggest the importance of hydrophobic interaction near the phenylchroman group of the most active compound in the training set. Halogen (except fluorine) substitution in orange region will enhance molecular hydrophobic field and increase the affinity of the compound. In addition, the introduction of hydrophilic groups (such as amide and amine) on the other side of the benzene ring (transparent gray) may increase α_1A_-AR antagonistic activity, whereas chlorine substitution in compound **32** may reduce the binding activity of α_1A_-AR, which is a representative of weak α_1A_-AR receptor binding.

The graphical interpretation of the field contributions of the H-bond properties is shown in [Figure 5(c)] (the H-bond acceptor field). Magenta isopleths (80%) encompass regions wherein hydrogen bond acceptor groups lead to improved α_1A_-AR antagonist activity; while areas encompassed by red isopleths (20%) should be avoided from hydrogen bond acceptor, which will result in impaired biological activity. Small red isopleths are inlaid in the purple region. The oxygen atom connected to the phenyl group may be an important factor to high activity of compound **20**. The introduction of a hydrogen bond acceptor in the area may further improve the activity.

**Figure 6:**
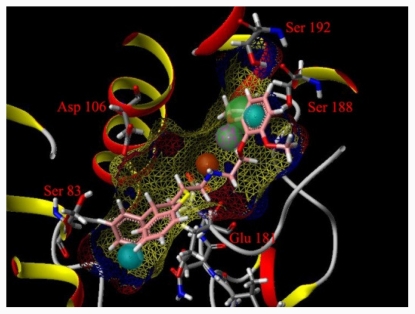
The binding pocket of α_1A_-AR homology model with compound **20** matching the pharmacophore.

3D structures of human α_1A_-AR homology models have been successfully developed by Li’s group based on the crystal structure of β_2_-AR [41]. This optimized homology model was retrieved from Protein Model Data Base (PMDB entry: PM0075211). The pharmacophore features were placed into the binding pocket of this model, as shown in Figure 6. According to previous reports [41,42], hydrophobic interactions may occur between the hydrophobic group of a ligand and pockets constituted by Trp102, Cys110, Tyr111, Ser158, Phe193, Phe288, Phe289 and Phe312. Asp106 seems to play a role in antagonist binding through strong electrostatic interactions with the protonated nitrogen atom. Ser188 and Ser192 may have H-bond effects with acceptor and donor features. In contrast to the most reported 3D-QSAR models mentioned in “Introduction”, GALAHAD was used in the present models to superimpose ligands based on pharmacophore molecular alignment. The flexible superimposition afforded by the current models more closely coincides with the receptor binding sites and reduces the likelihood of confusion arising from compounds belonging to different classes. The generated models suggested that electrostatic, hydrophobic, and hydrogen bonding interactions play important roles between ligands and receptors in the active site. The *q*^2^, *r^2^* *_pred_* and contours obtained from the present CoMFA and CoMSIA model show strong predictability and application and provide detailed information about the molecular features of the ligands, which will contribute to the antagonistic potency.

## 4. Conclusions

CoMFA and CoMSIA models were established based on a training set of 32 ligands using pharmacophore-based molecular alignment by GALAHAD. The statistical significance of the models was evaluated. The *q*^2^ values of the CoMFA and CoMSIA models are 0.840 (4 components) and 0.840 (3 components), respectively. These models also predict the biological activities of 12 ligands of α_1A_-AR antagonists in the test set.

In this study, GALAHAD is a useful pharmacophore alignment tool that can yield a good 3D-QSAR model for α_1A_-AR antagonists. The present results of the 3D-QSAR model provided a valuable tool for predicting the activities of novel α_1A_-AR antagonists and a basis upon which more active compounds can be derived by targeted structural modifications.

## Figures and Tables

**Table 1:** Structures of the training set molecules used in the 3D-QSAR study.

Series	Compd. No.	R_1_	R_2_	R_3_	X	*K_i_*(nM)	p*K_i_*
I	**1**	4-Cl	−OCH_3_	−OCH_3_	O	0.251	9.60
	**2**	2-CH_3_	−OCH_3_	−OCH_3_	O	1.58	8.80
	**3**	3-CH_3_	−OCH_3_	−OCH_3_	O	0.398	9.40
	**4**	4-CH_3_	−OCH_3_	−OCH_3_	O	1.99	8.70
	**5**	3-OCH_3_	−OCH_3_	−OCH_3_	O	1.99	8.70
	**6**	4-OCH_3_	−OCH_3_	−OCH_3_	O	1.99	8.70
	**7**	H	−OCH_3_	−OCH_3_	S	3.16	8.50
	**8**	H	−OC_2_H_5_	−OC_2_H_5_	O	3.16	8.50
	**9**	H	−OC_2_H_5_	−H	O	2.00	8.70

II	**10**	−Cl	−	-	-	441.29	6.36
	**11**	−CH	−	-	-	467.33	6.33
	**12**	−CN	−	-	-	170.88	6.77
	**13**	−Br	−	-	-	301.13	6.52
	**14**	−F	−F	-	-	108.10	6.97
	**15**	−Cl	−CH_3_	-	-	100.44	7.00
	**16**	−CH_3_	−CH_3_	-	-	95.42	7.02
	**17**	−CH_3_	−Cl	-	-	152.20	6.82
	**18**	−CN	−Cl	-	-	402.56	6.39
	**19**	−Cl	−F	-	-	86.16	7.06

III	**20**	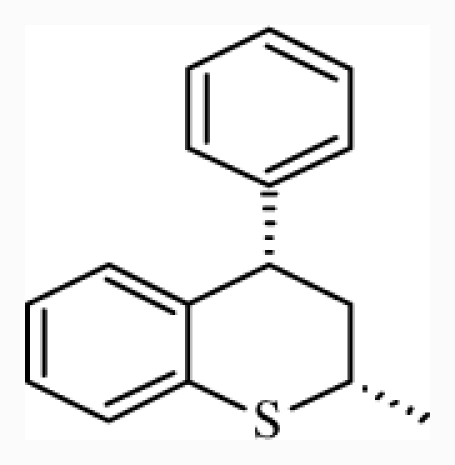	−OCH_3_	−OCH_3_	-	0.08	10.05
	**21**	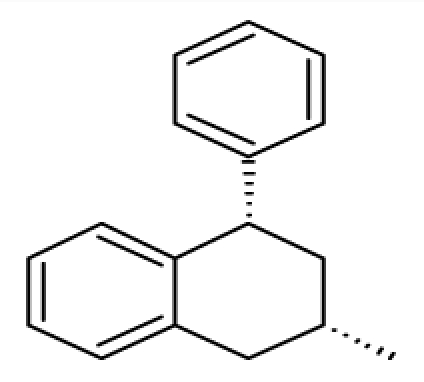	−OCH_3_	−OCH_3_	-	0.51	9.29
	**22**	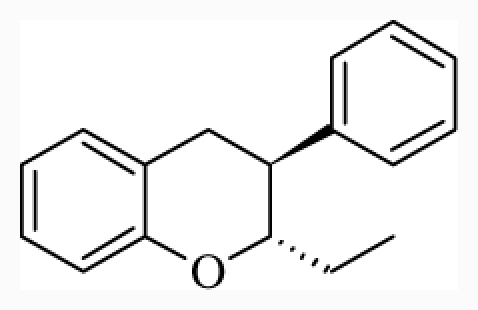	−OCH_3_	−OCH_3_	-	3.23	8.49

IV	**23**	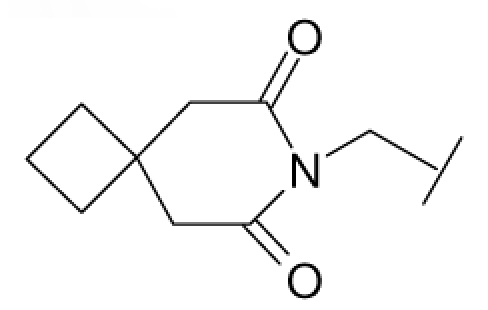	2-Cl	5-Cl	-	95.68	7.02
	**24**	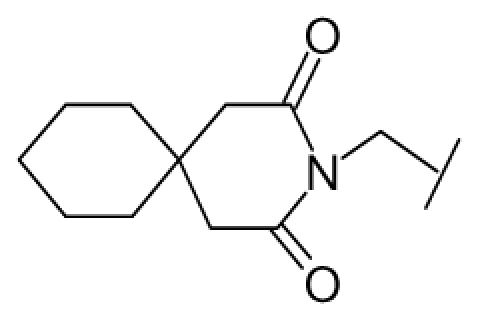	2-Cl	5-Cl	-	264.40	6.58
	**25**	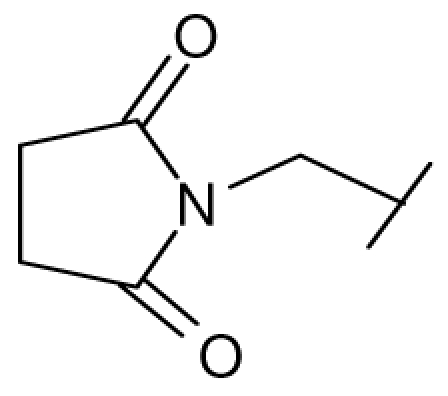	2-Cl	5-Cl	-	73.57	7.13
	**26**	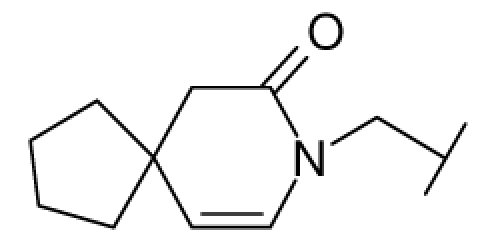	2-OCH_3_	-	-	23.4	7.63
	**27**	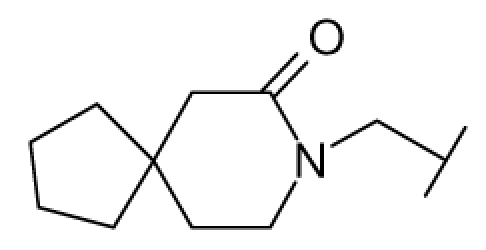	2-OCH_3_	-	-	43.54	7.36
	**28**	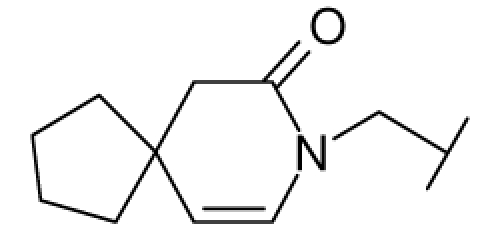	2-Cl	5-Cl	-	21.77	7.66
	**29**	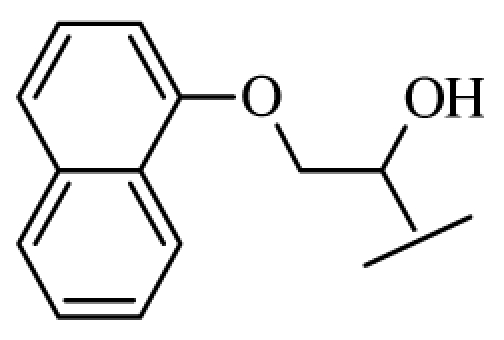	2-OCH_3_	-	-	5.88	8.23
	**30**	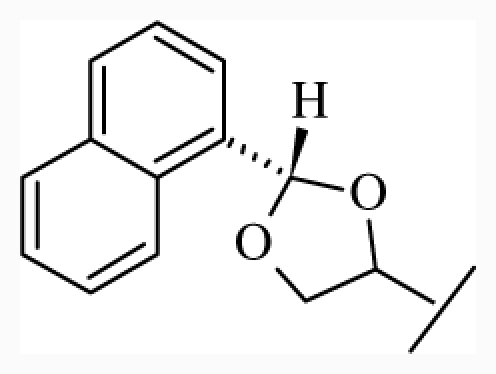	2-OCH_3_	-	-	7.94	8.10
	**31**	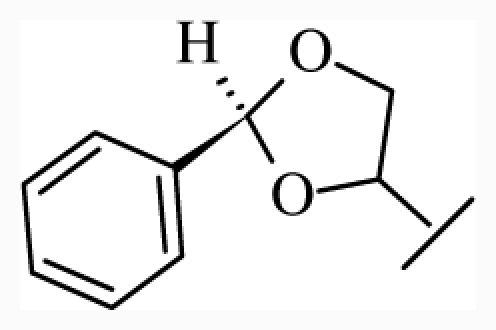	2-OCH_3_	-	-	28.84	7.54

V	**32**	-	-	-	-	629.04	6.20

**Table 2:** Structures of the test set molecules used in the 3D-QSAR study.

Series	Compd. No.	R_1_	R_2_	R_3_	X	*K_i_*(nM)	p*K_i_*
I	**33**	2-Cl	−OCH_3_	−OCH_3_	O	1.41	8.85

II	**34**	−Br	−Br	-	-	91.25	7.04
	**35**	−Cl	−I	-	-	383.60	6.42

III	**36**	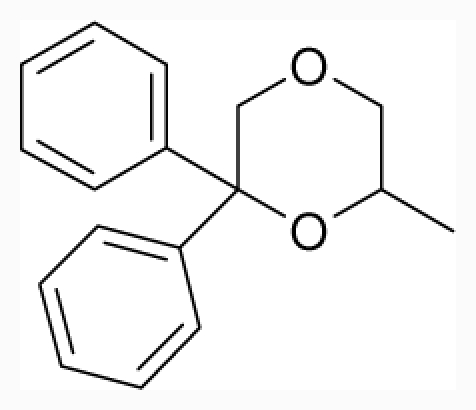	−OCH_3_	-	-	27.54	7.57
	**37**	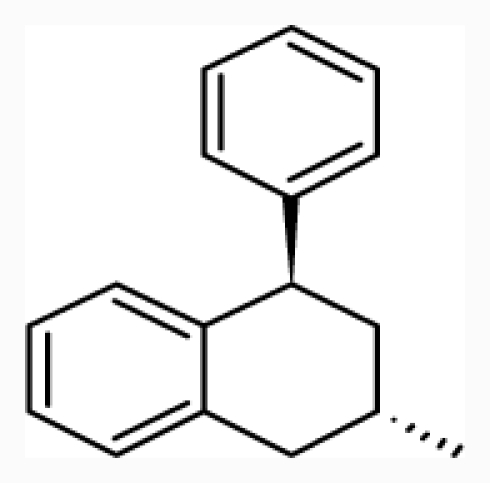	−OCH_3_	−OCH_3_	-	2.34	8.63
	**38**	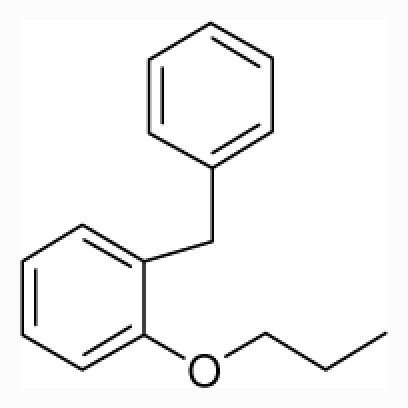	−OCH_3_	−OCH_3_	-	0.40	9.40
	**39**	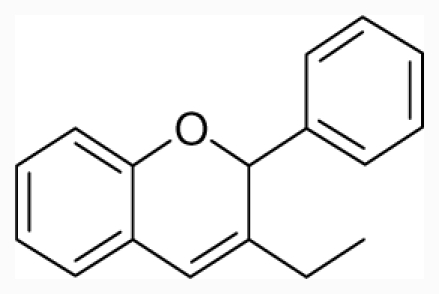	−OCH_3_	−OCH_3_	-	72.44	7.14

IV	**40**	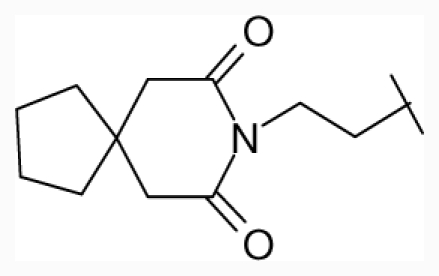	−Cl	−Cl	-	2.28	8.64
	**41**	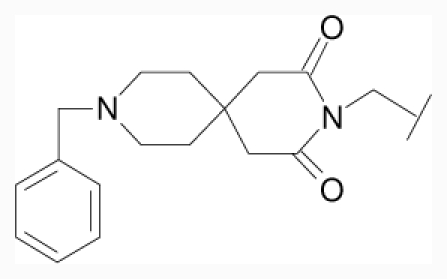	−OCH_3_	-	-	235.52	6.63
	**42**	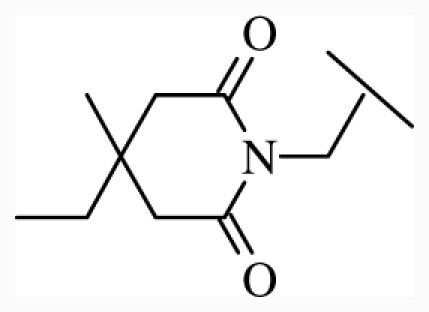	−Cl	-Cl	-	47.86	7.32
	**43**	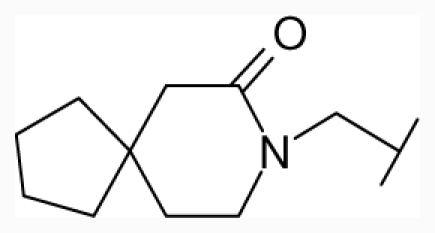	−Cl	-Cl	-	33.93	7.47

V	**44**	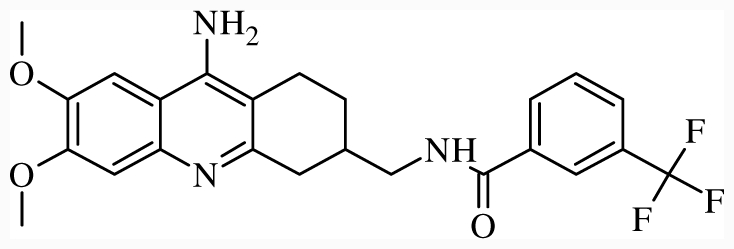				31.62	7.50

**Table 3:** Statistical analysis of CoMFA and CoMSIA models

Parameters a	CoMFA	CoMSIA b				
		A	B	C	D	E
Optimal PLS component	4	3	3	3	2	3
*q^2^*	0.840	0.874	0.866	0.842	0.856	0.840
*S_cv_*	0.476	0.407	0.419	0.456	0.427	0.459
*r^2^*	0.988	0.980	0.982	0.977	0.961	0.975
*SEE*	0.128	0.160	0.154	0.174	0.222	0.180
*F*	555.64	469.24	510.31	394.83	357.74	370.67
*r^2^_cv_*	0.837					0.864
*Fractions*						
Steric	0.460	0.104	0.203	0.153	0.133	
Electrostatic	0.540	0.217	0.386	0.297	0.264	0.347
Hydrophobic		0.212	0.410	0.336		0.399
Donnor		0.183			0.360	
Acceptor		0.285		0.214	0.243	0.254

*r^2^_pred_*	0.694	0.646	0.581	0.576	0.663	0.671

a *q_2_*, leave-one-out cross-validation correlation coefficient; *S_cv_*, leave-one-out cross-validated standard error; *r^2^*, conventional correlation; *SEE*, standard error of estimate; *F*, *F*-test value; *r*^2^ _cv_, conventional correlation of group cross-validation;

b CoMSIA model calculated using different field combinations. A, all fields; B, Steric, electrostatic and hydrophobic fields; C, Steric, electrostatic hydrophobic and acceptor fields; D, Steric, electrostatic donor and acceptor fields; E, electrostatic hydrophobic and acceptor fields.

**Table 4:** Experimental and cross-validated/predicted biological affinities and residuals obtained by the CoMFA and CoMSIA (model E) for 32 compounds in the training set and 12 compounds in the test set.

Compd. No.	p*K*_i_ (exp.)	p*K*_i_ (pred.)		Δp*K*_i_^a^	

		CoMFA	CoMSIA	CoMFA	CoMSIA
*Training set*					
**1**	9.60	9.620	9.561	−0.0197	0.0388
**2**	8.80	8.743	8.896	0.0572	−0.0957
**3**	9.40	9.410	9.273	−0.0103	0.1265
**4**	8.70	8.593	8.680	0.1072	0.0201

**5**	8.70	8.659	8.687	0.0412	0.0127
**6**	8.70	8.649	8.777	0.0505	−0.0771
**7**	8.50	8.510	8.611	−0.0103	−0.1113
**8**	8.50	8.440	8.676	0.0602	−0.1755
**9**	8.70	8.689	8.843	0.0108	−0.1432
**10**	6.36	6.505	6.694	−0.1453	−0.3342
**11**	6.33	6.385	6.474	−0.0548	−0.1435
**12**	6.77	6.652	6.716	0.1176	0.0540
**13**	6.52	6.559	6.605	−0.0385	−0.0847
**14**	6.97	6.923	6.901	0.0472	0.0688
**15**	7.00	7.113	7.035	−0.1129	−0.0352
**16**	7.02	6.880	6.690	0.1404	0.3303
**17**	6.82	6.793	6.656	0.0275	0.1644
**18**	6.39	6.475	6.641	−0.0852	−0.2509
**19**	7.06	7.011	7.024	0.0494	0.0363
**20**	10.05	10.126	9.768	−0.0759	0.2821
**21**	9.29	9.294	9.181	−0.0042	0.1095
**22**	8.49	8.601	8.751	−0.1107	−0.2609
**23**	7.02	6.871	6.661	0.1492	0.3588
**24**	6.58	6.590	6.691	−0.0096	−0.1111

**25**	7.13	7.177	7.202	−0.0472	−0.072
**26**	7.63	7.653	7.404	−0.0229	0.2259
**27**	7.36	7.365	7.290	−0.0055	0.0698
**28**	7.66	7.697	7.423	−0.0371	0.2370
**29**	8.23	8.275	8.179	−0.0451	0.0512
**30**	8.10	8.070	8.265	0.0305	−0.1652
**31**	7.54	7.576	7.631	−0.0364	−0.0906
**32**	6.20	6.217	6.235	−0.0173	−0.0351

*Test set*					
**33**	8.85	8.587	9.010	0.2629	−0.1603
**34**	7.04	6.766	6.613	0.2737	0.4265
**35**	6.42	6.669	6.653	−0.2490	−0.2334
**36**	7.57	8.514	8.370	−0.9437	−0.800
**37**	8.63	8.174	8.723	0.4563	−0.0931
**38**	9.40	9.804	9.128	−0.4037	0.2719

**39**	7.14	7.916	7.652	−0.7758	−0.5117
**40**	8.64	7.996	7.506	0.6440	1.1341
**41**	6.63	6.584	6.848	0.0461	−0.2182
**42**	7.32	7.794	6.801	−0.4744	0.5191
**43**	7.47	6.974	7.079	0.4955	0.3908
**44**	7.50	7.341	7.852	0.1589	−0.3518
*r^2^_pred_*^b^		0.694	0.671		

a Δp*K*_i_ is defined as p*K*_i_,_experimental_ − p*K*_i_,_cross-validated/predicted_;

b Predictive correlation coefficient of the test set is defined as *r^2^* *_pred_* = (SD – PRESS)/SD.
